# Supplementary material for: FastqPuri: high-performance preprocessing of RNA-seq data
Source: BMC Bioinformatics. 2019 May 3;20:226. doi: 10.1186/s12859-019-2799-0 (PMC6500068; doi:10.1186/s12859-019-2799-0)
Supplement: Supplementary file 2 — Archive of FastqPuri. Archive containing all files needed to install and run FastqPuri v1.0.6. Date stamp March 22, 2019. (GZ 47,819 kb) [file 12859_2019_2799_MOESM2_ESM.gz › FastqPuri-1.0.6/html/struct__fa__data.html]

FastqPuri: \_fa\_data Struct Reference


FastqPuri

Public Attributes |
List of all members

\_fa\_data Struct Reference

stores sequences of a fasta file
More...

`#include <fa_read.h>`

Collaboration diagram for \_fa\_data:

[legend]

|  |  |
| --- | --- |
| Public Attributes | |
| uint64\_t | nlines |
|  | |
| int | nentries |
|  | |
| int | linelen |
|  | |
| uint64\_t \* | entrylen |
|  | |
| Fa\_entry \* | entry |
|  | |

## Detailed Description

stores sequences of a fasta file

## Member Data Documentation

## ◆ entry

Fa\_entry\* \_fa\_data::entry

Array with fasta entries (see Fa\_entry)

## ◆ entrylen

uint64\_t\* \_fa\_data::entrylen

Array containing the length of the entries

## ◆ linelen

int \_fa\_data::linelen

Line length of the \*fa file entries

## ◆ nentries

int \_fa\_data::nentries

Number of entries in \*fa file

## ◆ nlines

uint64\_t \_fa\_data::nlines

Number of lines in \*fa file

---

The documentation for this struct was generated from the following file:

- include/fa\_read.h


---

Generated on Mon Mar 19 2018 23:42:01 for FastqPuri by  

 1.8.14
